# Technological innovations at the onset of the Mid-Pleistocene Climate Transition in high-latitude East Asia

**DOI:** 10.1093/nsr/nwaa053

**Published:** 2020-03-29

**Authors:** Shi-Xia Yang, Fa-Gang Wang, Fei Xie, Jian-Ping Yue, Cheng-Long Deng, Ri-Xiang Zhu, Michael D Petraglia

**Affiliations:** Key Laboratory of Vertebrate Evolution and Human Origins of Chinese Academy of Sciences, Institute of Vertebrate Paleontology and Paleoanthropology, Chinese Academy of Sciences, Beijing 100044, China; Center for Excellence in Life and Paleoenvironment, Chinese Academy of Sciences, Beijing 100044, China; Department of Archaeology, Max Planck Institute for the Science of Human History, Jena 07745, Germany; Hebei Provincial Institute of Cultural Relics, Shijiazhuang 050031, China; Hebei Provincial Institute of Cultural Relics, Shijiazhuang 050031, China; Department of History, Anhui University, Hefei 230039, China; State Key Laboratory of Lithospheric Evolution, Institute of Geology and Geophysics, Chinese Academy of Sciences, Beijing 100029, China; College of Earth and Planetary Sciences, University of Chinese Academy of Sciences, Beijing 100049, China; State Key Laboratory of Lithospheric Evolution, Institute of Geology and Geophysics, Chinese Academy of Sciences, Beijing 100029, China; College of Earth and Planetary Sciences, University of Chinese Academy of Sciences, Beijing 100049, China; Department of Archaeology, Max Planck Institute for the Science of Human History, Jena 07745, Germany; Human Origins Program, National Museum of Natural History, Smithsonian Institution, Washington DC 20560, USA; School of Social Science, The University of Queensland, Brisbane QLD 4072, Australia

**Keywords:** early hominins, behavioural adaptations, technological innovations, Mid-Pleistocene Climate Transition

## Abstract

The interplay between Pleistocene climatic variability and hominin adaptations to diverse terrestrial ecosystems is a key topic in human evolutionary studies. Early and Middle Pleistocene environmental change and its relation to hominin behavioural responses has been a subject of great interest in Africa and Europe, though little information is available for other key regions of the Old World, particularly from Eastern Asia. Here we examine key Early Pleistocene sites of the Nihewan Basin, in high-latitude northern China, dating between ∼1.4 and 1.0 million years ago (Ma). We compare stone-tool assemblages from three Early Pleistocene sites in the Nihewan Basin, including detailed assessment of stone-tool refitting sequences at the ∼1.1-Ma-old site of Cenjiawan. Increased toolmaking skills and technological innovations are evident in the Nihewan Basin at the onset of the Mid-Pleistocene Climate Transition (MPT). Examination of the lithic technology of the Nihewan sites, together with an assessment of other key Palaeolithic sites of China, indicates that toolkits show increasing diversity at the outset of the MPT and in its aftermath. The overall evidence indicates the adaptive flexibility of early hominins to ecosystem changes since the MPT, though regional abandonments are also apparent in high latitudes, likely owing to cold and oscillating environmental conditions. The view presented here sharply contrasts with traditional arguments that stone-tool technologies of China are homogeneous and continuous over the course of the Early Pleistocene.

## INTRODUCTION

Pleistocene stone-tool industries, particularly those from China, have traditionally been considered a homogenous and long-lasting technological tradition. Hallam Movius [1] famously compared stone-tool industries in Eastern Asia to those of the West, leading him to conclude that the Eastern toolkits were unstandardized, monotonous and unimaginative, thereby indicating that the toolmakers exhibited some level of ‘cultural retardation’ [2]. Though Movius’s views have been heavily critiqued by archaeologists [3–5], there is a persistent view that there were indeed long periods of technological stasis in China [6,7] ranging over a period of a million years or more. Lithic analysts working in Eastern Asia tend to categorize stone-tool assemblages found across the Pleistocene as simple, small-tool technologies and typable under terms such as ‘small-tool traditions’, ‘Chopper-Chopping tool industries’ and Mode I assemblages [8,9]. Moreover, archaeologists have tended to view the adaptations of hominins in Eastern Asia as conservative, suggesting that early human populations were passive actors on Pleistocene landscapes, exhibiting few, if any, novel traits and flexible behaviours for coping with environmental change. Consistently with this view, hominin occupations in Eurasia are considered short-term and non-permanent [10–13] and especially challenging for populations in high-latitude regions, such as northern China, which exhibited strong seasonal variability [14]. Moreover, hominins are thought to have maintained generalized adaptive strategies to particular grassland–woodland ecosystems over the long term and over vast parts of Asia, thereby suggesting that species such as *Homo erectus* exhibited low levels of behavioural plasticity [15].

In contrast to such characterizations, early hominins that moved across Asia displayed a remarkable level of adaptive and behavioural flexibility, evidenced by their presence in China by 2.1 Ma [16] and their expansion across a large geographic range and into a variety of high- and low-latitude ecosystems [17,18]. Indeed, it has been argued that hominins in diverse ecosystems of northern China between ∼1.66 and ∼1.36 Ma display increased levels of adaptability to high environmental diversity and variability, setting the stage for the expansion of populations into new climatic and biotic zones [19]. In addition, recent technological analyses of Chinese lithic assemblages across the Pleistocene are beginning to demonstrate the presence of significant variability in technology, diversity in toolmaking methods and changes in tool types and raw-material-selection patterns [9,20,21]. Hominins occupying Eastern Asia ∼800 thousand years ago (ka) demonstrate adaptive and technological flexibility, as evidenced by the manufacture of large cutting tools soon after a meteorite impact and forest burning [22]. Innovations in stone-tool reduction and the manufacture of finely retouched tools in Early Pleistocene assemblages have been suggested to be the consequence of responses to environmental variability [9,20,21], although this relationship has not been examined in any level of detail.

**Figure 1.**
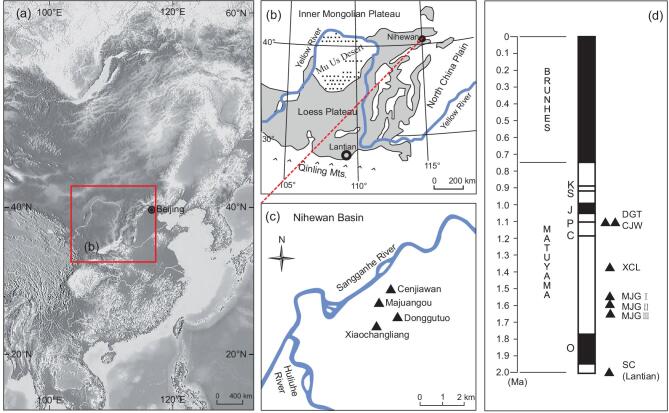
Nihewan Basin, China. (a) The Nihewan Basin, located in northern China. (b) The Yellow River is a major river system in north China. The Qinling Mountains are the traditional dividing line between north and south China. The Nihewan Basin occurs at the northern edge of the Loess Plateau. (c) The location of key Nihewan Early Pleistocene sites mentioned in the text: Cenjiawan (CJW), Majuangou (MJG), Donggutuo (DGT) and Xiaochangliang (XCL). (d) Palaeomagnetic chronology of key archaeological sites in the Nihewan Basin and the earliest site of Shangchen (SC, Lantian) within the framework of the geomagnetic polarity timescale [25,26]. K, Kamikatsura; S, Santa Rosa; J, Jaramillo; P, Punaruu; C, Cobb Mountain; O, Olduvai.

To assess the degree to which early stone-tool-using hominins modified their tool-manufacturing behaviours during the Early Pleistocene in Eastern Asia, we examined three well-known lithic sites from the Nihewan Basin in North China (Fig. 1a−c and Supplementary Note 1). The Nihewan Basin was selected for this study as it contains a long and well-developed fluviolacustrine sedimentary sequence with well-preserved archaeological sites of all stages of the Pleistocene, especially in the Early Pleistocene [23,24] (Fig. 1d and Supplementary Fig. 1). The chronology of the archaeological sites was established by correlation with the recognized magnetic polarities in the sedimentary sequence [18,23] and in accordance with the geomagnetic polarity timescale [25,26]. Here, we conducted a technological assessment of the Cenjiawan site, including a new analysis of lithic refits, alongside inter-assemblage comparisons of Xiaochangliang and Donggutuo, thereby providing unprecedented information about lithic assemblage formation between ∼1.4 and 1.1 Ma (Fig. 1c and d, and Supplementary Notes 1−4).

## RESULTS

### Stone-tool-knapping skills recorded in the Cenjiawan assemblage

Cenjiawan was first excavated in the 1980s, with a total of 1625 lithic artefacts and 257 mammal fossils retrieved [27–29]. Magnetostratigraphic dating results showed that the Cenjiawan artefact layer is just posterior to the Punaruu geomagnetic excursion [30], which has an ^40^Ar/^39^Ar age of 1.105 ± 0.005 Ma [26], with Donggutuo dating to about the same age (Fig. 1d). The lithic assemblages of Cenjiawan have been subject to previous lithic refitting studies [28,31,32], providing unique information about lithic manufacturing methods for Early Pleistocene assemblages of Eastern Asia. Yet, previous publications provide limited information about the lithic assemblages and knapping sequences, without wider exploration of their evolutionary implications. Here, we present a renewed study of the Cenjiawan lithic assemblage and the refitting groups, with the aim to examine details of stone-tool-knapping sequences to assess hominin technological skills and planning abilities (Supplementary Note 3).

The lithic assemblages at Cenjiawan were produced mainly by freehand hard-hammer percussion (FHHP) with rare examples of bipolar artefacts. The lithic artefacts are small in size, with 60% of the artefacts measuring <20 mm, suggesting minimal transport and sorting by fluvial processes [33,34]. The proportion of small artefacts and the high percentage of refit sets with all stages of stone-tool reduction indicate on-site knapping activities by hominins. The lithic artefacts are composed of cores, flakes and flake fragments, accounting for 41.28% of the assemblage, whereas shatter, angular fragments and unflaked pebbles in a variety of sizes account for 54.27% of the assemblage (Supplementary Table 1). A total of 54 retouched artefacts were identified, including scrapers, denticulates, borers and notches, accounting for 3.34% of the lithic assemblage.

**Figure 2.**
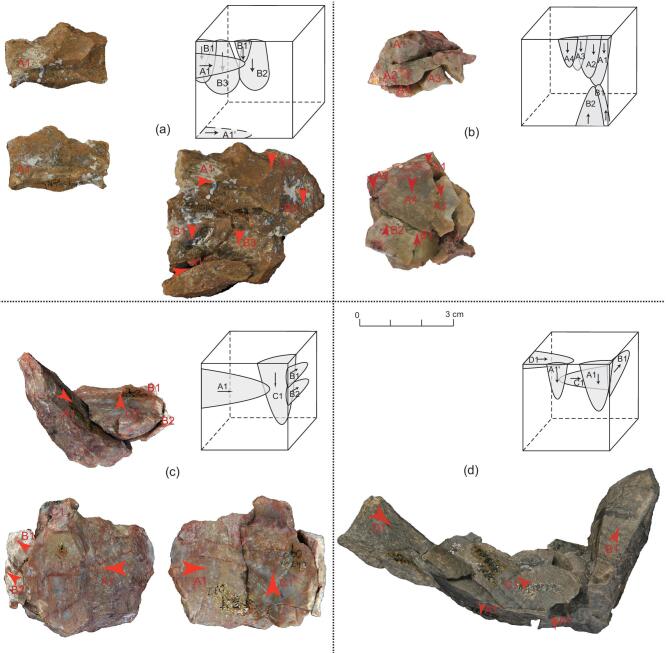
Refitting groups from Cenjiawan showing reduction sequences. (a) Refitting group a (six items), a two-directional (vertical) refitting group on a chert nodule. (b) Refitting group b (six items), a two-directional (opposite) refitting group on a chert nodule. (c) Refitting group c (four items), a three-directional refitting group on a chert nodule. (d) Refitting group d (eight items), a four-directional refitting group on argillaceous siltstone.

Cenjiawan has been subject to an intensive programme of refitting, producing detailed information about stone-tool-reduction sequences and behavioural activities [28,31,32] (Fig. 2). However, in previous studies, detailed information on the refitted groups and metric results has not been reported. To understand the formation of lithic assemblages and reconstruct knapping sequences, the refitted pieces were studied in detail and diacritical schemes of flaking sequences were produced (Fig. 2). In this study, a total of 411 lithic artefacts are identified among 1616 pieces, comprising 105 refitted groups and accounting for 25.43% of the lithic assemblage. The refits are mainly represented by the production of freehand percussion, including cores (40.98%), whole flakes (42.2%) and flake fragments (22.53%) (Supplementary Table 1). A high percentage (29.63%) of refits were represented by retouched pieces. Few of the bipolar pieces could be refitted (*n* = 2, 11.11%). A relatively high percentage of the refits are from angular fragments (54.91%) and shatter (13.11%). In addition to refits, there were 88 conjoins (i.e. distal to proximal conjoins, left-to-right conjoins) with 41 groups of conjoins represented by both flakes and tools (Supplementary Fig. 2).

The refitting groups ranged from high numbers (with up to 25-pieces refit) to low numbers (only 2 pieces) (Supplementary Table 2 and Supplementary Fig. 3). The groups with high numbers of refits provide detailed information about a dynamic process of reduction sequences, including information on the direction of flaking and on core rotation, thus allowing assessment of hominin planning procedures in order to obtain desired flake and tool products.

Among the refitting groups, bidirectional flaking procedures were observed in 25 cases (Fig. 2a and b). Figure 2a illustrates a refitted reduction sequence composed of six complete flakes and one retouched piece on a flake. This case illustrates an example where knappers obtained a number of flakes from two percussion directions, with a 90° turn in core rotation. Flake A1 was chosen from the group of flakes and finely retouched in order to produce a tip on the mid-section of a lateral edge. This type of retouched piece was not a unique find, suggesting that particular flake blanks were sought for producing tipped pieces (Supplementary Fig. 4). Figure 2b is another example of a bidirectional reduction sequence, composed of six refit flakes. In this case, the knapper used two opposing flake platforms, turning the core 180° to perform flaking. The resultant core was highly consumed based on its small size and the application of a series of small flake (<30 mm) removals.

Figure 2c illustrates a 3D reduction sequence, which is observed in 11 cases. The refit pieces in Fig. 2c show two changes in the direction of reduction, which is composed of four flakes. The first flaking sequence is represented by a whole flake (A1), which is later selected as a blank for toolmaking. Two flakes (B1, B2) were then removed from another platform and probably found to be undesirable, as they were short and without typical conchoidal flake attributes, probably on account of the presence of poor-quality material. The core was then rotated a third time (sequence C) in an attempt to obtain additional, desired flakes (e.g. Flake C1). The reduction sequence illustrated in Fig. 2c is a good example of how knappers avoided low-quality interstitial material, while seeking higher-quality chert, as noted at other Nihewan sites [20,21].

Four-directional flaking procedures were observed in three cases. Figure 2d illustrates a refitted reduction sequence composed of three complete flakes and two broken flakes. The first flaking sequence is represented by two short flakes (A1 and A1’). One thick flake (B1) was then removed from another platform. The core was then rotated a third time (sequence C) in an attempt to obtain additional flakes, while the flake was broken (C1 is a flake conjoined by two parts). After the removal of the broken flake (C1), the core was then turned to strike it in a fourth direction and D1 was removed. Flake D1 was broken into three parts. Although the centre portion of this reduction group was missing, the outer part indicates that the knapper attempted to choose suitable directions for flaking, changing direction in order to more fully consume the core.

Refit groups represented by only two to three flake refits (*n* = 63) are presented in the Supplementary Data. These refit groups mainly show single-direction flaking and bidirectional flaking owing to the low refit numbers (Supplementary Tables 2 and 3, and Supplementary Fig. 3). Given their incomplete nature, it is of course difficult to determine if they were from simple unidirectional production sequences or parts of more complex reduction groups.

### Technological comparisons of the Nihewan Basin assemblages

Reanalysis of the Cenjiawan lithic assemblages has provided new insights about stone-tool manufacture at ∼1.1 Ma. The refitting analysis indicated that the Cenjiawan knappers aimed to obtain as many flakes from cores as possible, changing the striking platform to achieve good results, thus showing some degree of forethought and planning in tool reduction. To understand how the Cenjiawan lithic assemblages compared to other Early Pleistocene sites in the Nihewan Basin, lithic data were obtained from Xiaochangliang (∼1.36 Ma) [20,35] and Donggutuo (∼1.1 Ma) [21,36] (Supplementary Tables 4−6 and Supplementary Notes 2 and 4). As comprehensive studies have recently been conducted on the lithic assemblages from these sites [20,21], a unique opportunity presented itself to understand lithic assemblage formation in the Nihewan Basin between ∼1.36 and 1.1 Ma.

**Figure 3.**
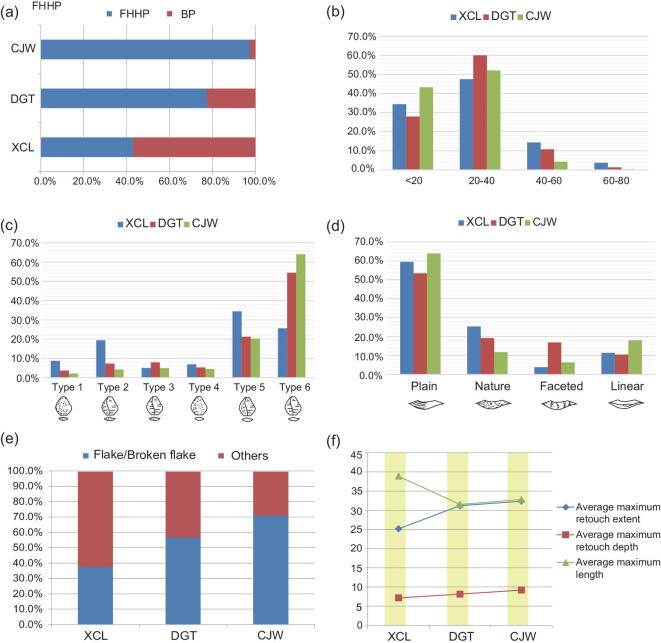
Lithic technology comparisons of three Early Pleistocene sites in the Nihewan Basin: Xiaochangliang (XCL), Donggutuo (DGT) and Cenjiawan (CJW). (a) The relative frequency of freehand hard-hammer percussion (FHHP) and bipolar percussion flakes. (b) Flake size ranges (in mm). (c) Flake attributes according to Toth types [37]. (d) Striking platform types on flakes. (e) Tools blank types. (f) Comparisons of average maximum retouch extent, retouch depth and tool length.

In all the three Nihewan sites, chert was exploited as the dominant raw material, ranging from 96% to 68% of the total lithic assemblage. FHHP and bipolar percussion were applied in all three sites, though with different frequency [20,21,27] (Fig. 3a). Cenjiawan had the highest percentage (97.37%) of hard-hammer products (i.e. cores, flakes, flake fragments), followed by Donggutuo (77.47%) and Xiaochangliang, the latter of which had a much lower percentage (43.06%) [20]. The bipolar technique was applied frequently (56.94%) at Xiaochangliang, and more rarely at Donggutuo (22.35%) and Cenjiawan (2.73%) [20,21] (Fig. 3a), indicating that more controlled flaking methods were applied in the younger sites (Supplementary Table 4).

Flake sizes were generally small at all three sites (Fig. 3b). Donggutuo and Cenjiawan had the highest frequency of small flakes; 95.39% of the flakes at Cenjiawan were <40 mm and no flake was >60 mm. At Xiaochangliang, a higher percentage of the flakes were >40 mm (Fig. 3b). Although small flakes were common in all three sites, the younger sites had a higher frequency of small flakes. The flake data generally correspond with core sizes, as cores from Xiaochangliang are larger in comparison to the younger sites (Supplementary Tables 5 and 6, and Supplementary Fig. 5). The production of smaller flakes from freehand percussion indicates well-controlled skills in working small nodules. The higher percentage of smaller cores relates to the efficient exploitation of raw materials as well. The refitting sets from Cenjiawan show that core reduction sequences (see the idealized schemes in the upper right-hand corner of Fig. 2) are similar to those from Xiaochangliang and Donggutuo, including the unidirectional, bidirectional, bifacial and multidirectional types [20,21]. Even though there are similar reduction sequences in the three sites, the flake attributes on the two later assemblages illustrate the dexterity of early hominins in obtaining desirable flakes from small rock nodules. Higher percentages of non-cortical flakes (types VI and V) [37] were recorded in Donggutuo and Cenjiawan (Fig. 3c). Type VI and V flakes account for 59% in Xiaochangliang, 76% in Donggutuo and 85% in Cenjiawan. The higher percentage of smaller flakes (types V and IV) at Cenjiawan and Donggutuo suggest more in-depth reduction and a continuous flaking of cores, whereas, at Xiaochangliang, the smaller assemblage likely results from roughing-out cores and the application of bipolar percussion [20]. Figure 3d shows that plain and natural platforms were more frequent in the somewhat older site of Xiaochangliang. In contrast, there was more preparation of striking platforms at Donggutuo and Cenjiawan in order to control flaking. Donggutuo had a relatively high percentage of faceted platforms, with Cenjiawan having the highest percentage of linear platforms. The linear platform type at Cenjiawan likely relates to low flake thicknesses, with flakes having an average maximum thickness of  7.15 mm. Both Donggutuo and Cenjiawan show that the knappers were skilful in producing smaller and thinner conchoidal flakes by hard-hammer percussion, though their flaking strategies differed somewhat. At Cenjiawan, knappers repeatedly changed core reduction directions to produce small and thin flakes. At Donggutuo, cores were sometimes prepared to work small nodules. This included the preparation of platforms on ‘wedge-shaped’ cores with an aim to produce microblade-like forms [21].

In each of the three sites, high-quality and standardized retouched pieces were produced (Supplementary Figs 6 and 7), contrary to suggestions that there was no shaping of tools in these lithic assemblages [9]. The retouched pieces of the three sites are small in size and show regular and repeated retouching on the same margins of the blanks producing distinct features (Supplementary Fig. 7). Among the tool types were forms that can be classed as scrapers, denticulates, borers, notches and pointed tools. Compared to Cenjiawan and Donggutuo, the Xiaochangliang assemblage had a lower percentage of retouched tools (2.5%). Retouch was simpler in Xiaochangliang and in Cenjiawan and Donggutuo, though more standardized tool forms were recognizable, such as borers and concave-edged scrapers (Supplementary Table 7). Retouched tools were commonly manufactured on flake blanks at Cenjiawan and Donggutuo, whereas, at Xiaochangliang, bipolar splinters and angular broken pieces predominate (Fig. 3e), corresponding to evidence of more controlled flaking at the younger sites. Figure 3f reinforces this pattern, demonstrating that, at Cenjiawan and Donggutuo, the average maximum retouch extent and maximum retouch depth are longer, as opposed to the Xiaochangliang tools.

Inter-assemblage comparisons between Xiaochangliang, Cenjiawan and Donggutuo, together with the refitting study at Cenjiawan, indicate that technological skills increase at ∼1.1−1.0 Ma. The lithic assemblages at Cenjiawan and Donggutuo show increasing levels of control in flaking procedures despite small clast size and raw-material flaws, some degree of planning in the knapping process to produce desired flake blanks and the application of retouch to produce standardized tools. The wedge-shaped cores from Donggutuo show parallel flake scars and partly prepared platforms, indicating some degree of planning and foresight to produce the desired end products [21,38]. While utilizing the same type of raw materials, the Cenjiawan and Donggutuo hominins demonstrate some significant changes in lithic reduction methods in comparison to Xiaochangliang, including the application of more control and skilful conchoidal flaking methods, suggesting the efficient utilization of clasts and planning to obtain desired blanks. Our findings are supported by the recent analysis of the Madigou assemblages, dating to ∼1.2 Ma, which show innovations in toolmaking, such as an increased emphasis on bipolar flaking, strong raw-material preferences, the presence of a variety of retouched tools and the shaping of some large tools [39].

## DISCUSSION

**Figure 4.**
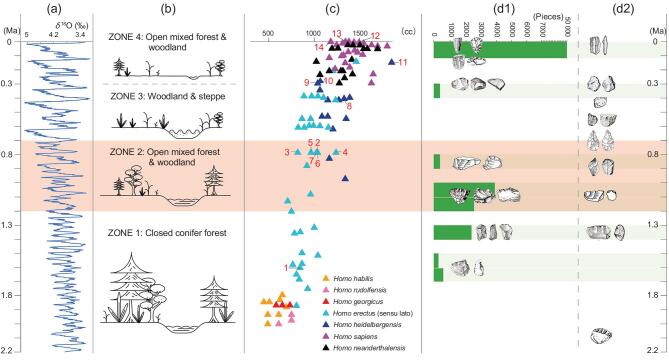
Synthesis of ecological, biological and lithic data from the Early to Late Pleistocene (the light red horizontal bar denotes the MPT). (a) Global climate change based on the benthic δ^18^O stack LR04 [42]. (b) Vegetation history of the North China Plain based on pollen analysis [53]. (c) Hominin brain-size estimates for Africa and Eurasia [69] (numbers represent key fossils from China, Supplementary Table 7; *Homo heidelbergensis is considered to include Middle Pleistocene populations in Africa and Europe, while the Asian fossils at this time are difficult to group, thus the term mid-Pleistocene *Homo* [62] is sometimes used). (d1) Lithic changes through the time in the Nihewan Basin, showing the frequency of lithic artefacts across different time periods (dark-green bars). (d2) Key lithic changes across China as discussed in the main text.

To evaluate technological change over the long term and the relative skills of toolmakers at ∼1.1−1.0 Ma, it is instructive to examine the Nihewan sites in relation to lithic assemblages from earlier and later sites of China (Fig. 4). At the 2.1-Ma-old site of Shangchen (Lantian) [14] (Fig. 4d2), FHHP was applied to cores along a single platform in order to produce a few flakes. Likewise, at Majuangou III, the oldest site in the Nihewan Basin (Fig. 4d1), a relatively simple core-flake-reduction sequence was identified at ∼1.66 Ma [18,29]. Though retouched pieces were present at Majuangou, these were not regular in shape, having retouching typically confined to small areas on a single edge. The Shangchen and Majuangou III lithic assemblages contrast with those from Xiaochangliang, dating to ∼1.36 Ma, as somewhat increased technological flexibility is indicated by the use of both bipolar and freehand reduction techniques, including the presence of retouched pieces that show more regularity in form [20]. Therefore, we suggest that the Early Pleistocene lithic assemblages at Shangchen and Majuangou III show relatively straightforward percussion techniques to obtain flake products, whereas, by ∼1.36 Ma, increased toolmaking skills are evident, just prior to the technological innovations witnessed at ∼1.0−1.1 Ma at Cenjiawan and Donggutuo (Fig. 4d1).

The technological innovations evidenced at ∼1.1−1.0 Ma in the Nihewan Basin correspond to the early stages of the MPT (Fig. 4a), which is characterized by an alteration of the length and intensity of glacial–interglacial cycles and by the periodicity of high-latitude climate oscillations, changing from 41- to 100-kyr cycles [40−43]. In a number of geological records, particularly those from Eastern Asia, the 100-kyr cycles date to between ∼1.2 and ∼0.7−0.6 Ma [40−43] (Supplementary Note 5). This variability was accompanied by a series of global or regional palaeoclimatic and palaeoenvironmental changes, such as increases in aridity and monsoonal intensity and decreases in sea-surface temperatures in the North Atlantic and tropical-ocean upwelling regions [40]. The complex environmental fluctuations in northern parts of East Asia included strengthened winter monsoons, weakened summer monsoons and strengthened aridity in high-latitude areas [44−48].

Early Pleistocene hominin populations of northern China, including the Nihewan Basin, would have experienced climatic changes and environmental shifts caused by the MPT. This included a stepwise southerly migration of the Mu Us desert, C4 plant expansions in the Loess Plateau, changes in vegetation communities moving towards more open mixed forests and woodlands (Fig. 4b) and the extinction and extirpation of a number of mammalian species [44,45,47−50]. In the Nihewan Basin, environmental changes are recorded at Donggutuo and Cenjiawan, with a shift towards more open grass steppe with mixed forests and woodlands [51,52]. At Donggutuo and at the fossiliferous site of Shanshenmiaozui, the number of grassland mammalian fauna increased at ∼1.2−1.1 Ma, whereas warm and humid adapted species went extinct (e.g. *Leptobas stenometopon*, *Hesperotherium*) [53]. During the onset of the MPT, however, changes in temperatures and their oscillations were not as extreme as in later periods, perhaps stimulating innovations in early hominin toolkits and signalling behavioural responses to ecosystem changes at Cenjiawan and Donggutuo. The production of a variety of retouched tool types (e.g. scrapers, denticulates, notches, borers, pointed tools) at these sites indicates diverse activities (i.e. scraping, cutting, piercing), perhaps linked with the processing of animal and plant foods as found in other early archaeological sites of Africa and Asia [54,55]. The ability of hominins to adapt to environmental conditions brought on by the MPT are not only recorded in high-latitude China, as archaeologists have also related climatic shifts in this period with dispersal events, novel behaviours and changes in lithic technology [12,13,56].

From ∼1.1−1.0 Ma and towards the end of MPT, site numbers and artefact densities declined dramatically in the Nihewan Basin with only rare finds at Maliang and, between ∼0.8 and 0.4 Ma, there is a gap in the archaeological record [24], coincident with longer and harsher cold periods (Fig. 4d1). Though sites are present in China between ∼0.7 and 0.3 Ma (Fig. 4d2), they are either in the southern latitudes (e.g. Chenjiawo, Dali) or in caves (e.g. Zhoukoudian), with some evidence for fire use [57−60]. Towards the end of the MPT, at ∼0.9−0.8 Ma, the first signs of large cutting-tool assemblages appear at Yunxian (Xuetangliangzi) and in the Bose Basin [22,61], and, in the case of the latter, novel stone-tool-using behaviours are thought to relate to ecosystem changes [22]. After the MPT, archaeological data are poorly represented in the Nihewan Basin and in North China, perhaps as a reflection of high seasonality in colder climates [14], whereas, in lower latitudes of central and southern China, Middle Pleistocene hominin sites are present [58,62,63], suggesting the presence of habitable environments and the sustained survival of hominin populations. After a long hiatus of occupation, the oldest Middle Pleistocene site of the Nihewan Basin is Hougou, dated to ∼395 ka, indicating the reappearance of hominins and showing the use of unidirectional core reduction and the irregular production of retouched tools [64,65], representing a novel lithic assemblage, and perhaps indicating the loss of earlier technological innovations.

On the whole, the lithic industries between 1.1 and 0.3 Ma across China show considerable diversity and variation in core reduction strategies, ranging from relatively simple to more advanced flaking procedures (i.e. cores with few flake removals, wedge-shaped cores) and in tool production (large bifacial and unifacial cutting tools, frequency of retouched flakes). While innovations are present, there is no clear sign of a consistent, unilinear technological evolution in toolmaking, suggesting that behavioural and adaptive responses to environmental and ecological conditions are responsible for lithic assemblage formation.

A question may be posed as to whether technological innovation and adaptive changes correspond with biological evolution, such as hominin brain-size increase. Figure 4c illustrates that hominin brain sizes generally increase from the Early to Middle Pleistocene on an intercontinental level [66−69]. Although brain-size increases are recorded after the MPT, considerable variations occur in the Middle Pleistocene [69−71]. In China, a trend for increasing brain size is notable between the Gongwangling *Homo erectus* cranium, dated to 1.61 Ma [72], with a cranial capacity of 780 cc [70], and finds at Zhoukoudian, with cranial capacities ranging widely between 850 and 1225 cc at ∼0.77 Ma [70] (Supplementary Table 7). Unfortunately, however, no measurable fossil crania are available in China between the critical threshold between 1.61 and 0.77 Ma. It could be noted, however, that stone innovations in the Nihewan Basin at Cenjiawan and Donggutuo at ∼1.1−1.0 Ma do not necessarily coincide with larger brain sizes based upon cranial data outside China (Fig. 4c). It is difficult to correlate brain-size trends with changes in lithic technology, other than noting that large cutting-tool innovations at ∼0.8 Ma in southern China coincide with the larger brain sizes represented at Zhoukoudian, though no necessary connection can be drawn. It is also worthwhile to point out that selection for larger brain sizes is not indicated in the later Middle Pleistocene of China, as Dali is recorded as 1160 cc at 0.3−0.26 Ma and Hualongdong is recorded as 1150 cc at 0.3 Ma [73] (Supplementary Table 7). The most significant wholesale changes in lithic technology and material culture occur in the Late Pleistocene, when brain-size increases are recorded at Xuchang (1800 cc) at 125−105 ka and in the Zhoukoudian Upper Cave (1290−1500 cc) at ∼35 ka (Supplementary Table 8). Unfortunately, at this point in time, it remains difficult to disentangle the relationships between the MPT and biological and cultural evolution until additional fossils are discovered.

In sum, at ∼1.1 Ma, the inhabitants in the Nihewan Basin lived under a changeable and unstable environment, experiencing strengthened aridification [44,48]. As climatic variability produced ecological changes, including landscape alterations and faunal turnovers, novel technological innovations may have provided some selective benefits to early hominin populations in the Nihewan Basin. The unstable environmental conditions at the onset of the MPT provide a good example of the adaptive versatility of hominins in China during the Early Pleistocene, contrasting with the notion of long-lasting conservative behaviours and undifferentiated technologies across the Pleistocene. Yet, the increasingly harsh and oscillating climatic conditions of the MPT likely undermined sustained population in North China, illustrating that technological and cultural solutions did not always overcome environmental challenges.

## Supplementary Material

nwaa053_Supplemental_File
