# Supplementary material for: Technological innovations at the onset of the Mid-Pleistocene Climate Transition in high-latitude East Asia
Source: Natl Sci Rev. 2020 Mar 29;8(1):nwaa053. doi: 10.1093/nsr/nwaa053 (PMC8288396; doi:10.1093/nsr/nwaa053)
Supplement: nwaa053_Supplemental_File [file nwaa053_supplemental_file.docx]

**Supplementary Data**

**Technological Innovations at the Onset of the** **Mid-Pleistocene Climate Transition in High-Latitude East Asia**

Shi-Xia Yang^1,2,3*^, Fa-Gang Wang^4^, Fei Xie^4^, Jian-Ping Yue^5^, Cheng-Long Deng^6,7^, Ri-Xiang Zhu^6,7^, Michael D. Petraglia^3,8,9*^

1. Key Laboratory of Vertebrate Evolution and Human Origins of Chinese Academy of Sciences, Institute of Vertebrate Paleontology and Paleoanthropology, Chinese Academy of Sciences, Beijing 100044, China

2. Center for Excellence in Life and Paleoenvironment, Chinese Academy of Sciences, Beijing 100044, China

3. Department of Archaeology, Max Planck Institute for the Science of Human History, Jena 07745, Germany

4. Hebei Provincial Institute of Cultural Relics, Shijiazhuang 050031, China

5. Department of History, Anhui University, Hefei 230039, China

6. State Key Laboratory of Lithospheric Evolution, Institute of Geology and Geophysics, Chinese Academy of Sciences, Beijing 100029, China

7. College of Earth and Planetary Sciences, University of Chinese Academy of Sciences, Beijing 100049, China

8. Human Origins Program, National Museum of Natural History, Smithsonian Institution, Washington, D.C. 20560, USA

9. School of Social Science, The University of Queensland, Brisbane, Queensland 4072, Australia

Contents

Supplementary Notes3

Supplementary Note 1. The Nihewan Basin and surrounding area3

Supplementary Note 2. Samples and data collection4

Supplementary Note 3. Refitting analysis5

Supplementary Note 4. Dating results of Xiaochangliang and Donggutuo6

Supplementary Note 5. Timing of the Mid-Pleistocene transition (MPT)7

Supplementary Tables9

Supplementary Table 1. Lithic assemblage components and refitting information from Cenjiawan9

Supplementary Table 2. Overall count of refitting groups from Cenjiawan 9

Supplementary Table 3. Flaking direction among the refitted groups from Cenjiawan 10

Supplementary Table 4. Lithic assemblage components from Xiaochangliang (XCL), Donggutuo (DGT) and Cenjiawan (CJW)10

Supplementary Table 5. The dimensions of cores (mm) of the Xiaochangliang (XCL), Donggutuo (DGT) and Cenjiawan (CJW) 11

Supplementary Table 6 Statistical tests results of the cores’ maximum length from Xiaochangliang (XCL), Cenjiawan(CJW) and Donggutuo(DGT) 11

Supplementary Table 7. Tool types from Xiaochangliang (XCL), Donggutuo (DGT) and Cenjiawan (CJW)11

Supplementary Table 8. Dating estimates and endocranial capacity of fossil hominins from China12

Supplementary Figures 13

Supplementary Figure 1. The lacustrine deposits in the Nihewan Basin of North China 13

Supplementary Figure 2. Conjoined pieces at Cenjiawan13

Supplementary Figure 3. Examples of simple refitting groups among flakes at Cenjiawan.14

Supplementary Figure 4. A well-defined refitting group including a finely retouched tool from Cenjiawan14

Supplementary Figure 5. Cores sizes from Xiaochangliang(XCL), Donggutuo (DGT) and Cenjiawan(CJW) (in mm)15

Supplementary Figure 6. Tool types with diagnostic characteristics15

Supplementary Figure 7. Representative tool types from Xiaochangliang (XCL), Donggutuo (DGT) and Cenjiawan (CJW).16

Supplementary Figure 8. The plan and profile of some refitting groups at Cenjiawan16

Supplementary References17

**Supplementary Notes**

**Note 1.** **The Nihewan Basin and surrounding area**

The Nihewan Basin is located at the northeastern edge of the Loess Plateau and at the transition zone between the North China Plain and the Inner Mongolia Plateau (Figure 1). The basin contains up to 150 m of exposed deposits that accumulated in and around a weak to semi-saline palaeolake (Figure 1 and Supplementary Figure 1) [1], dating from the Late Pliocene [2-4] to the Middle Pleistocene, at ca. 420−260 ka [5-6], when the water body finally dried up.

There is no long, high-resolution record concerning changes in flora and fauna of the basin. In order to understand the ecological background of the Nihewan Basin over the long term, it is necessary to consult the geological and paleoenvironmental record of the surrounding region. A key, recent study of a drill core placed in the North China Plain revealed a long sequence of changes in paleovegetation patterns, which was compared against changes in large mammal communities [7]. This study illustrated climatic fluctuations during the Mid-Pleistocene climate transition (MPT) and linkages with ecosystems. Before 1.2 Ma, vegetation was mainly composed of alternating warm temperate forests and temperate forests; after 1.2 Ma, vegetation was mainly characterized by alternations in forests and grasslands [7]. The Mu Us Desert to the west of the Nihewan Basin also shows an episode of the expansion at about 1.2 Ma, and intensified aridification after 0.7 Ma [8-9]. Up to the onset of the Late Pleistocene, the region is characterized by a warm coniferous-broadleaved mixed forest with woody-grassland, as well as an increase in the extent of wetlands [7]. Pollen studies of the Nihewan Basin from key sections indicate that a relatively dry and cold desert grassland environment was present early on, as shown in the Nangou section from 2.5 to 2.0 Ma [10]. A well-developed forest was present until the transition to the MPT, with pollen evidence indicating the existence of subtropical plants, such as Sapindaceae*, Hymenophyllum sm.,* and *Lyodium sw.* [11]. During the MPT, at ca. 1.2-1.0 Ma, a stepwise intensification of aridification is recorded by the presence of grasses and herbs at Donggutuo [11-13]. The limited information available from the Nihewan Basin is generally consistent with the sequences established in the North China Plain.

For Figure 4, we gathered paleoclimatic and ecological information in and around the Nihewan Basin, including the benthic isotope data from ODP Site 1143 of the South China Sea and the pollen data of the drilling core from the North China Plain. Figure 4 illustrates the environmental background, assisting in interpretations about the context of changes in stone tool knapping through time.

## Note 2. Samples and data collection

Xiaochangliang, Donggutuo and Cenjiawan are key Early Pleistocene sites in the Nihewan Basin, and include ca. 5,000 lithic artefacts (Supplementary Table 4). Xiaochangliang (40º13'10"N, 114º39'44"E) was discovered in 1978 and later dated to ca. 1.36 Ma by magnetostratigraphy. In the current study, a total of 1184 lithic artefacts were included in the analyses. Donggutuo (40º13'22"N, 114º40'11"E) is situated in the eastern margin of the Nihewan Basin and was identified in 1981. The Donggutuo cultural layers date to ca. 1.1 Ma by magnetostratigraphy. In the current study, 2315 pieces artefacts were included in the analyses. Cenjiawan (40º13'21"N, 114º40'17"E) was identified in 1984, and dated to ca. 1.1 Ma by magnetostratigraphy. In the current study, 1383 artefacts were analyzed. The lithic artefacts from the three sites have been systematically studied and compared using the same methods, making it possible to have a comprehensive understanding of inter-assemblage lithic variability through the creation of a single comparative database.

The key attributes of the main classes of artefacts, i.e., cores, flakes, and shaped tools, have been compared in each of the Nihewan lithic assemblages. The classification systems used in East African Oldowan assemblages has been followed [14-18], while taking the characteristics of the Nihewan lithic assemblages and raw materials into account. The Xiaochangliang, Donggutuo and Cenjiawan sites contain retouched tools which are significance for learning about tool use and the skills of early hominins. Several attributes (e.g., size charateristics of retouched pieces; blank retouch types; tool types; the average maximum retouch extent and the average maximum retouch depth) were taken into account for inter-site comparison.

## Note 3. Refitting study

The Cenjiawan site, with a relative high refitting rate of stone artefacts (33.4 %, or 462 pieces of 1383 artefacts), was selected in order to conduct a study of stone tool knapping skills and to assess the cognitive ability of early humans. Previous refitting studies [19-22] provided important background information on knapping sequences. Most of the refitting groups are from an area of 5m^2^, including the excavation of units M, N, O, P and Q (each unit is 1mx1m). The maximum distance in vertical depth of refits is ca. 16 cm (Supplementary Figure 5).

In describing the refitting artefact sets, we follow Cziesla [23] in distinguishing between refits and conjoins. Refits are sets of artefacts split from each other by controlled fracture (e.g., a flake whose ventral side joins to the surface of a core or the dorsal surface of another flake). Conjoins are fragments of formerly whole artefacts broken by forces other than conchoidal fracture (i.e., natural flaws, bending fractures). The most common form of conjoins are distal and proximal fragments of the same flake, or the left and the right fragments of the same flake. In our study, we checked the previously refit artefacts and obtained 105 refitted groups and 41 conjoined groups. To reconstruct knapping sequences, we used the refitted group data, examining the type of the exploitation methods that the prehistoric knappers employed.

**Note 4. Dating results of Xiaochangliang and Donggutuo**

Magnetostratigraphic dating of the fluvio-lacustrine sequence in the Nihewan Basin has been a significant undertaking, permitting evaluation of the timing of basin infilling and dating of associated mammalian faunas and archaeological sites [2,24-25]. Magnetostratigraphic dating of long stratigraphic sequences requires assumptions about sedimentation rates, hence anchoring of age estimates are always best achieved through the application of multiple chronometric methods. Though debated and subject to change, overall, magnetochronological findings indicate that the earliest archeological sites of the fluvio-lacustrine Nihewan Beds date from the post-Olduvai Matuyama chron (~1.66 Ma for MJG-III) to the middle Brunhes chron (ca. 395 ka for Hougou) [26].

The site of Xiaochangliang has four estimated ages. In the current study, we cited the result of 1.36 Ma by Zhu et al. (2001) [25]. Here we summarise other age estimates and their potential problems:

- The age of 1.48 Ma is from Ao et al. (2010) [27] was obtained through stratigraphic correlation between the Dachangliang (also named Xiantai) section and the Xiaochangliang section. Thus, the age estimate of 1.48 Ma for the Xiaochangliang section is obtained indirectly.
- The age estimate of 1.26 Ma was obtained on the basis of sedimentological comparisons and sediment grain size distributions from the Xiaodukou and Xiaochangliang sections [28]. In Li et al.’s paper [28], magnetostratigraphic studies were carried out on the Xiaodukou and Donggutuo sections, not on the Xiaochangliang section. Therefore, the age of 1.26 Ma is an indirect age estimate.
- The age of 1.67 Ma was obtained based on the presence of certain types of mammalian fauna, as well as unpublished information about the magnetostratigraphy, informally provided by other scientists [29]. According to palaeomagnetic results obtained using cryogenic magnetometers, e.g., Zhu et al. (2001) [25] and Li et al. (2008) [28], the Xiaochangliang cultural layer is not located just above the Olduvai normal subchron. Thus, its age should be significantly younger than the age of the upper boundary of Olduvai (1.77 Ma).

Taking into account the above-mentioned age estimates for the Xiaochangliang site, a range from 1.67 Ma to 1.26 Ma has been suggested [28-29]. However, we maintain that the age of 1.36 Ma obtained by Zhu et al. (2001) is the best age estimate for the Xiaochangliang site, and fits well in the synthetic diagram from well-dated sections in the Nihewan Basin [25].

Magnetostratigraphic dating of the Donggutuo cultural layers has been conducted by several scholars since 1980s’ [30-32], and these dating projects have obtained similar results. According to the latest dating work [28,32], the age of the Donggutuo cultural layer is prior to the onset of Jaramillo normal subchron, which has been dated at 1.053±0.006 Ma [33] or 1.072 Ma [34]. The detailed magnetostratigraphic work by Wang et al. (2005) described the short interval with a possible geomagnetic excursion (E3) within the pre-Jaramillo Matuyama reverse chron (and also within the Donggutuo artifact layer) [32]; this may be correlated to the Punaruu normal geomagnetic excursion, which has an ^40^Ar/^39^Ar age determination of 1.105 ± 0.005 Ma [33]. This lends further support to the contention that the Donggutuo Palaeolithic site has an age of about 1.1 Ma [32].

**Note 5. Timing of the Mid-Pleistocene climate transition (MPT)**

Milanković’s theory suggested that orbitally induced summer insolation change at high-latitude region of the Northern Hemisphere played a key role in driving the ice cycles during the Pleistocene [35]. A significant shift occurred around the end of Early Pleistocene, and afterward into the Middle Pleistocene, with the length and intensity of the glacial-interglacial cycles significantly increasing, and with the dominant periodicity of high-latitude climate oscillations changing from 41 kye to 100 kyr. These significant changes in climate is named the mid-Pleistocene climate transition (MPT). Since its first recognition by Pisias and Moore (1981) [36], there have been extensive discussions about timing, duration, and mechanisms [37-43].

The timing of MPT is of importance when discussing the topic on human evolution. The MPT began at about 1.25 Ma [39] or about 1−0.8 Ma [44-47] and terminated at about 0.7–0.6 Ma [46-47]. Correlation between the grain size records of Chinese loess-paleosol sequence and the marine oxygen isotope records revealed a change in the dominant climatic periodicity from 41 kyr to 100 kyr at about 1–0.8 Ma [44].

**Supplementary Tables**

**Supplementary Table 1.** Lithic assemblage components and refitting information from Cenjiawan

| **Category** | **Current study** | **%** | **refitted** | **% refitted** |
| --- | --- | --- | --- | --- |
| Core (Freehand) | 61 | 3.78 | 25 | 40.98 |
| Flake (Freehand) | 282 | 17.45 | 119 | 42.20 |
| Flake fragment | 324 | 20.05 | 73 | 22.53 |
| Bipolar Core and splinter | 18 | 1.11 | 2 | 11.11 |
| Retouched pieces | 54 | 3.34 | 16 | 29.63 |
| Shatter | 681 | 42.14 | 81 | 13.11 |
| Angular fragment | 173 | 10.71 | 95 | 54.91 |
| Pebble/unmodified pieces | 23 | 1.42 | - | - |
| TOTAL | 1616 | 100 | 411 | 25.43 |

**Supplementary Table 2.** Overall count of refitting groups from Cenjiawan

| **No. of pieces per refitting group** | **No. of refitting groups** | **Total** |
| --- | --- | --- |
| 2 | 42 | 84 |
| 3 | 21 | 63 |
| 4 | 13 | 52 |
| 5 | 9 | 45 |
| 6 | 8 | 48 |
| 7 | 4 | 28 |
| 8 | 1 | 8 |
| 9 | 3 | 27 |
| 10 | 2 | 20 |
| 11 | 1 | 11 |
| 25 | 1 | 25 |
| -- | 105 | 411 |

**Supplementary Table 3.** Flaking direction among the refitted groups from Cenjiawan

| **The direction no.** | **No. of groups** | **% of the refitted group** |
| --- | --- | --- |
| One direction | 66 | 62.86 |
| Two directions | 25 | 23.81 |
| Three directions | 11 | 10.48 |
| Four directions | 3 | 2.86 |
| TOTAL | 105 | 100 |

**Supplementary Table 4.** Lithic assemblage components from Xiaochangliang (XCL), Donggutuo (DGT) and Cenjiawan (CJW)

| **Lithic class** | **XCL** | **%** | **DGT** | **%** | **CJW** | **%** |
| --- | --- | --- | --- | --- | --- | --- |
| Core (Freehand) | 43 | 3.63 | 245 | 10.58 | 61 | 3.78 |
| Flake (Freehand) | 160 | 13.51 | 380 | 16.41 | 282 | 17.45 |
| Flake fragment | 129 | 10.90 | 300 | 12.96 | 324 | 20.05 |
| Bipolar Core and splinter | 439 | 37.08 | 269 | 11.62 | 18 | 1.11 |
| Retouched pieces | 45 | 3.80 | 228 | 9.85 | 54 | 3.34 |
| Shatter | 292 | 24.66 | 558 | 24.11 | 681 | 42.14 |
| Angular fragment | 76 | 6.42 | 335 | 14.47 | 173 | 10.71 |
| Pebble/ unmodified pieces | -- | -- | -- | -- | 23 | 1.42 |
| TOTAL | 1184 | 100 | 2315 | 100 | 1616 | 100 |

**Supplementary Table 5.** Cores from Xiaochangliang (XCL), Donggutuo (DGT) and Cenjiawan (CJW) (in mm).

|  | **Length** | | **Width** | | **Thickness** | |
| --- | --- | --- | --- | --- | --- | --- |
|  | **Mean** | **Std.D** | **Mean** | **Std.D** | **Mean** | **Std.D** |
| XCL | 52.7 | 28.8 | 47.7 | 23.9 | 35.2 | 19.2 |
| DGT | 37.80 | 16.43 | 49.53 | 20.29 | 36.18 | 19.97 |
| CJW | 33.07 | 13.9 | 40.44 | 23.04 | 29.74 | 33.1 |

**Supplementary Table 6.** Statistical tests results of the cores’ maximum length from Xiaochangliang (XCL), Cenjiawan(CJW) and Donggutuo(DGT)

| **The** **compared pair** | **Student's t test results (P value)** | **Mann-Whitney U test (P value)** |
| --- | --- | --- |
| CJW-DGT | 0.1038 | 0.0678 |
| CJW-XCL | <0.01 | <0.01 |
| DGT-XCL | <0.05 | <0.05 |

**Supplementary Table 7.** Tool types from Xiaochangliang (XCL), Donggutuo (DGT) and Cenjiawan (CJW)

| **Tool type** | **XCL** | **%** | **DGT** | **%** | **CJW** | **%** |
| --- | --- | --- | --- | --- | --- | --- |
| Scraper | 32 | 71.11 | 176 | 77.19 | 38 | 70.37 |
| Borer | 3 | 6.67 | 10 | 4.38 | 4 | 7.41 |
| Notch | 4 | 8.88 | 17 | 7.46 | 4 | 7.41 |
| Denticulate | 3 | 6.67 | 5 | 2.19 | 2 | 3.7 |
| Pointed tool | -- | -- | 3 | 1.32 | -- | -- |
| Unidentified | 3 | 6.67 | 17 | 7.46 | 6 | 11.11 |
| Total | 45 | 100 | 228 | 100 | 54 | 100 |

**Supplementary Table 8.** Dating estimates and endocranial capacity of fossil hominins from China

| **No.** | **Hominid cranial** | **Age (Ma)** | **Brain size (cc)** | **Dating ref.** | **Brain size ref.** |
| --- | --- | --- | --- | --- | --- |
| 1 | Gongwangling | 1.61 | 780 | [48] | [49] |
| 2 | Zhoukoudian II | 0.77±0.08 | 1030 | [50] | [49] |
| 3 | Zhoukoudian VI | 0.77±0.08 | 850 | [50] | [49] |
| 4 | Zhoukoudian X | 0.77±0.08 | 1225 | [50] | [49] |
| 5 | Zhoukoudian XI | 0.77±0.08 | 1015 | [50] | [49] |
| 6 | Zhoukoudian XII | 0.77±0.08 | 1030 | [50] | [49] |
| 7 | Zhoukoudian III | 0.77±0.08 | 937.5 | [50] | [49] |
| 8 | Hexian | 0.41 | 1305 | [51] | [49] |
| 9 | Hualongdong | 0.30 | 1150 | [52] | [49] |
| 10 | Dali | 0.281+0.046/-0.041 | 1160 | [53] | [49] |
| 11 | Xuchang | 0.125-0.105 | 1800 | [54] | [54] |
| 12 | Upper Cave 101 | 0.035-0.033 | 1500 | [55] | [49] |
| 13 | Upper Cave 102 | 0.035-0.033 | 1380 | [55] | [49] |
| 14 | Upper Cave 103 | 0.035-0.033 | 1290 | [55] | [49] |

**Supplementary Figures**


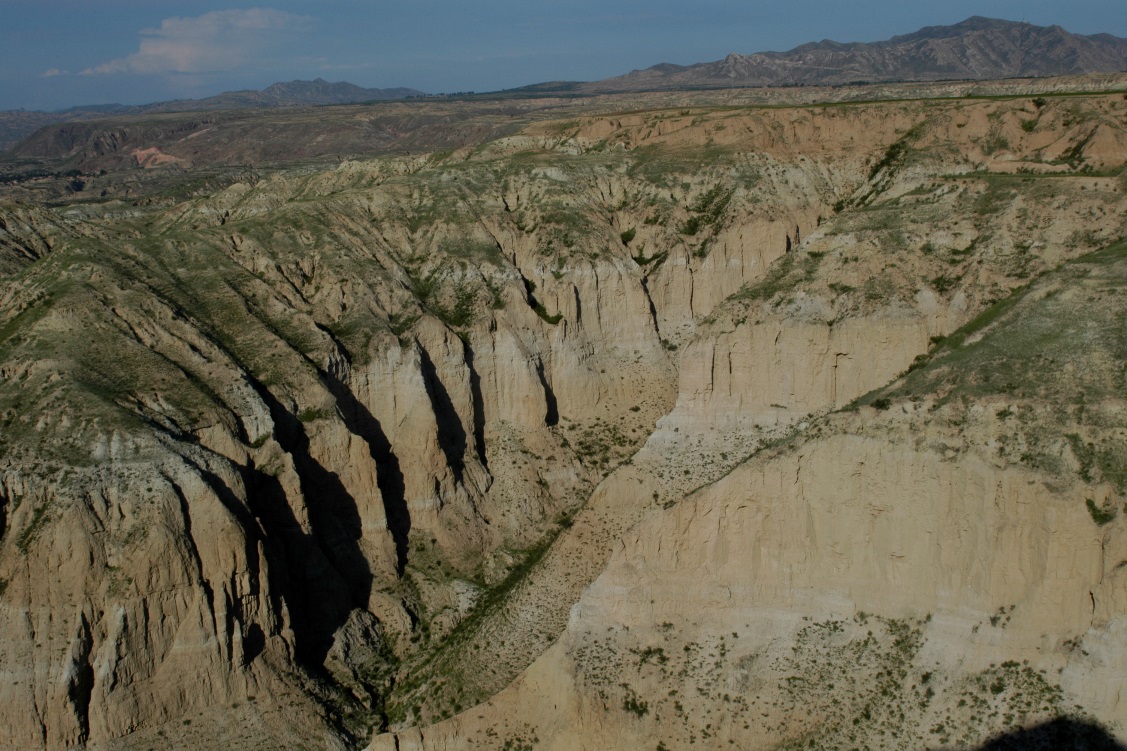


**Supplementary Figure 1. Fluvio-lacustrine deposits in Nihewan Basin of North China**


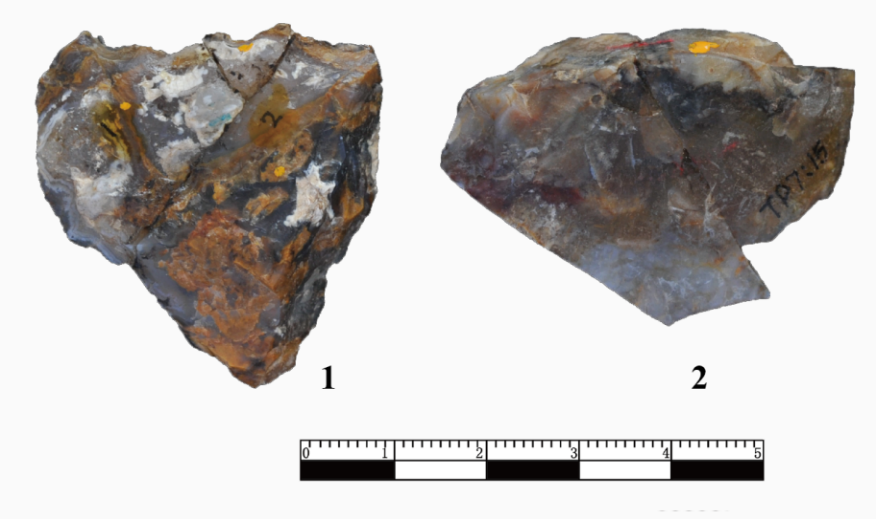


**Supplementary Figure 2.** Conjoined pieces from Cenjiawan


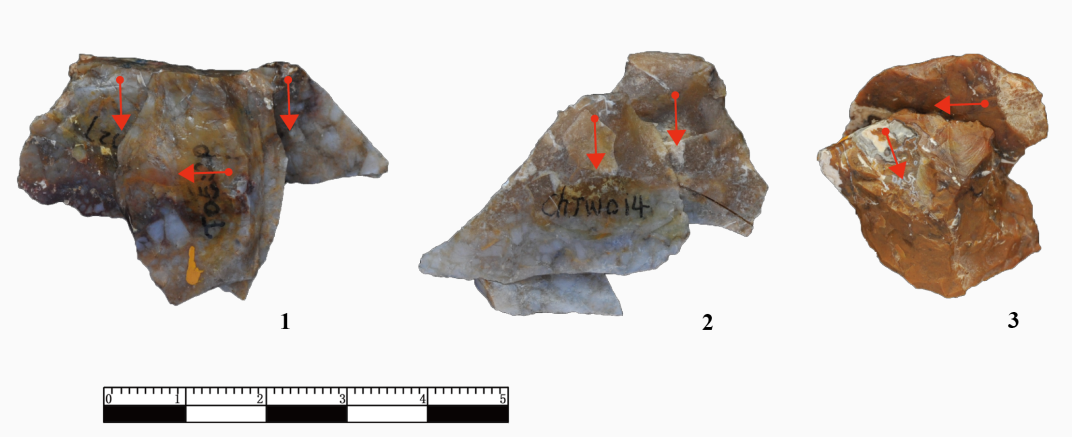


**Supplementary Figure 3.** Examples of simple refitting groups among flakes at Cenjiawan. Flake refits in these groups provide information about reduction sequences and core exploitation methods.


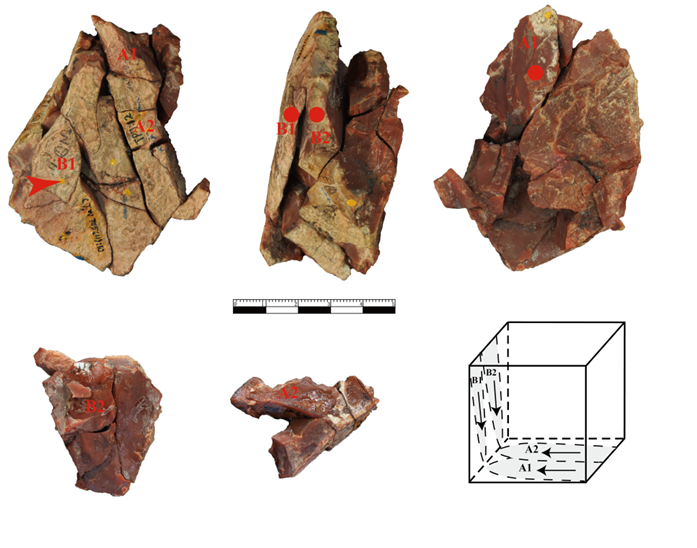


**Supplementary Figure 4.** A well-defined refitting group including a finely retouched tool from Cenjiawan. This refitting group consists of 35 pieces, including a retouched piece, broken flakes and broken flake fragments. This refitting group is a two directional reduction sequence. No whole flakes were produced. A long flake was used to produce a borer. The tip of the borer was made at the middle portion of the flake, along a single lateral edge.

**Supplementary Figure 5.** Cores sizes from Xiaochangliang (XCL), Donggutuo (DGT) and Cenjiawan (CJW)


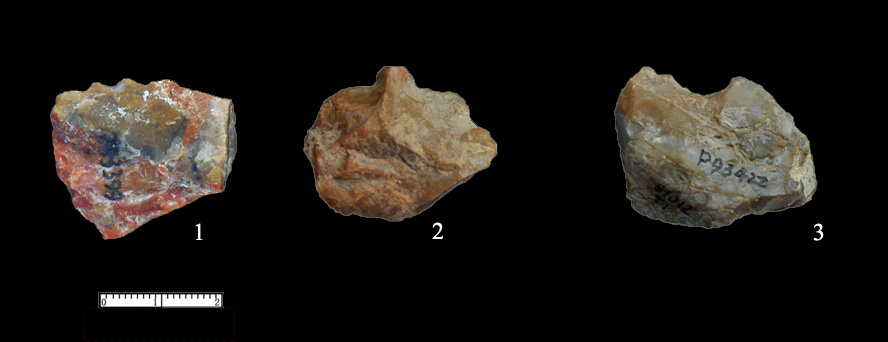


**Supplementary Figure 6.** Tool types with diagnostic characteristics (1. Denticulate; 2. Borer; 3. Notch)


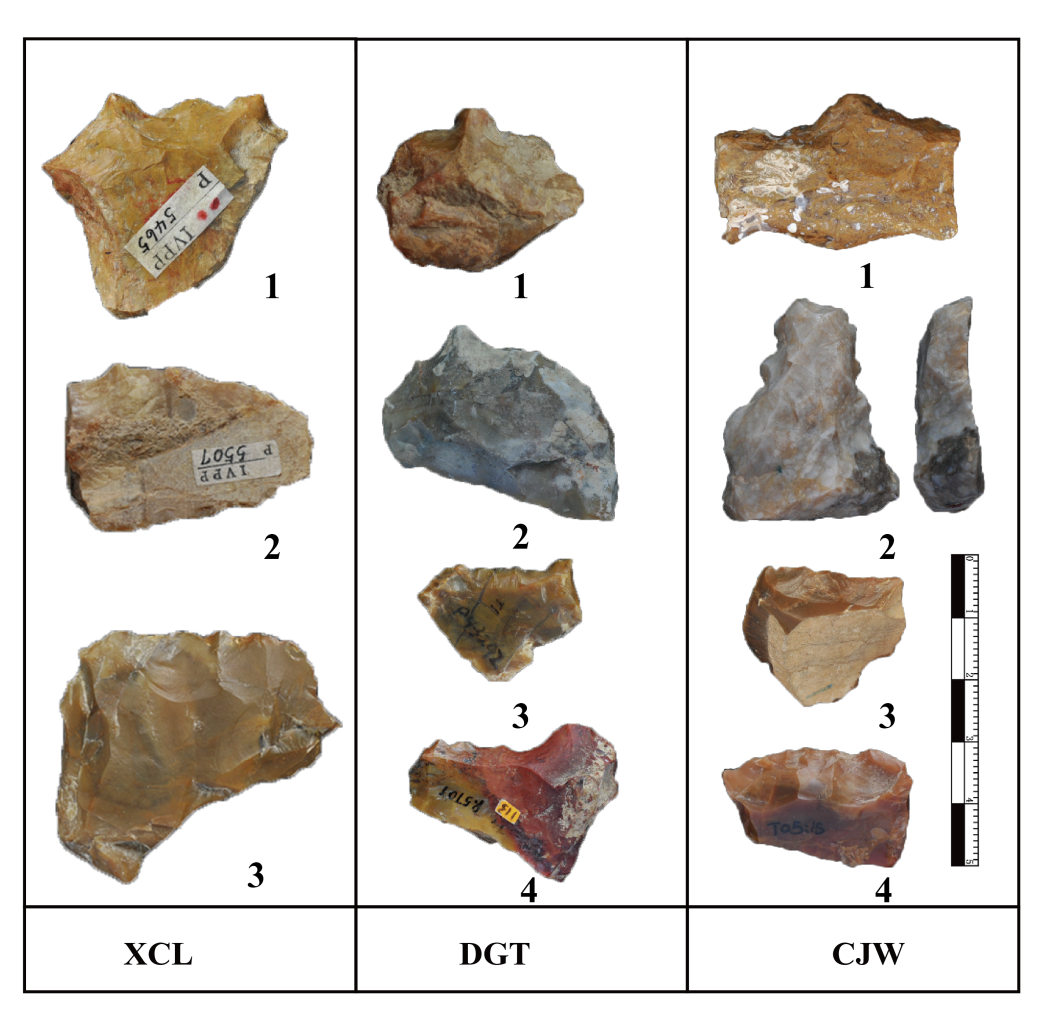


**Supplementary Figure 7.** Representative tool types from Xiaochangliang (XCL), Donggutuo (DGT) and Cenjiawan (CJW). Nos. 1, 2 from DGT and No. 1 from CJW are intentionally retouched borer tips. Scrapers with long and strait retouched ends were identified in the three assemblages (XCL-2, 3; DGT-3, 4; CJW-2, 3, 4); in the latter two the retouch scars were more invasive and regular.


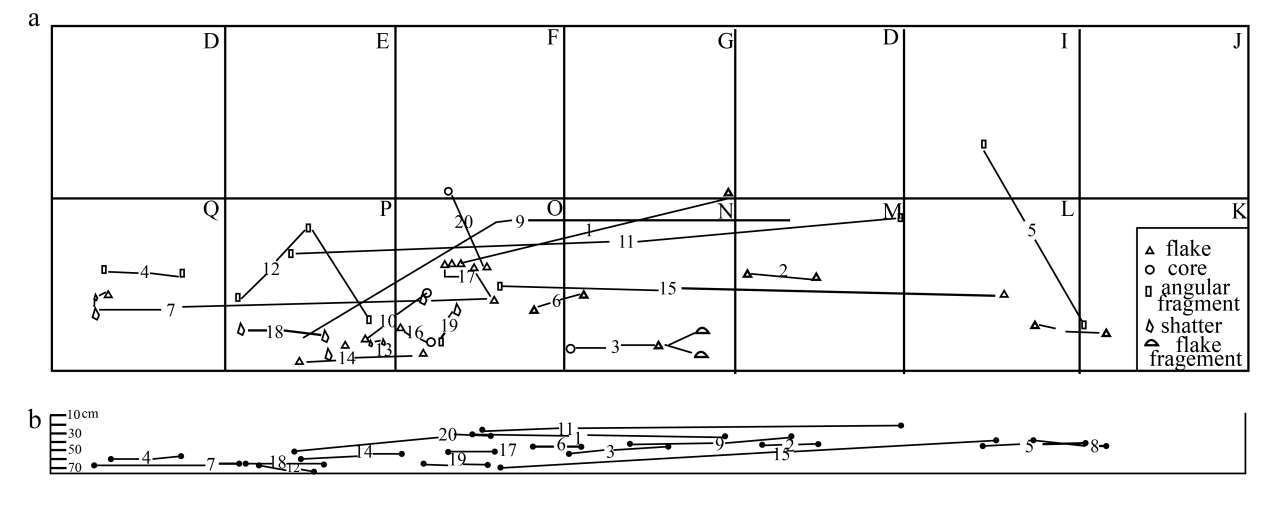


**Supplementary Figure 8.** The plan and profile of refitting groups at Cenjiawan. Most of the refitting groups are from an area of 5 m^2^, including excavation units M, N, O, P and Q (each unit is 1x1 m). The maximum distance in vertical depth of refits is ca. 16 cm [21].

**Supplementary References**

1. Li RQ, Qiao JG and Qiu WL *et al*. Soluble salt deposit in the Nihewan beds and its environmental significance. *Sci China Earth Sci* 2000; **43**: 464–79.
2. Deng CL, Zhu RX and Zhang R *et al*. Timing of the Nihewan formation and faunas. *Quat Res* 2008; **69**: 77–90.
3. Deng CL, Hao QZ and Guo ZT *et al*. Quaternary integrative stratigraphy and timescale of China. *Sci China Earth Sci* 2019; **62**: 324–48.
4. Liu P, Yue F and Liu JQ *et al*. Magnetostratigraphic dating of the Shixia red sediments and implications for formation of Nihewan paleo-lake, North China. *Quat Sci Rev* 2018; **193**: 118–28.
5. Zhao H, Lu Y and Wang C *et al*. ReOSL dating of aeolian and fluvial sediments from Nihewan Basin, northern China and its environmental application. *Quat Geochronol* 2010; **5**: 159–63.
6. Nian XM, Zhou LP and Yuan BY. Optically stimulated luminescence dating of terrestrial sediments in the Nihewan Basin and its implication for the evolution of ancient Nihewan Lake. *Quat Sci* 2013; **31**: 404–13.
7. Zhou XY, Yang JL and Wang SQ *et al*. Vegetation change and evolutionary response of large mammal fauna during the Mid-Pleistocene transition in temperate northern East Asia. *Palaeogeogr Palaeoclimatol Palaeoecol* 2018; **505**: 287–94.
8. Ding ZL, Derbyshire E and Yang SL *et al*. Stepwise expansion of desert environment across northern China in the past 3.5 Ma and implications for monsoon evolution. *Earth Planet Sci Lett* 2005; **237**: 45–55.
9. Yang SL, Ding F and Ding ZL. Pleistocene chemical weathering history of Asian arid and semi-arid regions recorded in loess deposits of China and Tajikistan. *Geochim Cosmochim Acta* 2006; **70**: 1695–709.
10. Zhou KS, Liang XL and Yan FH *et al*. Some remarks on the Nangou cold period based on the analysis of the pollen from the Nihewan stratum. *Chin J Geol* 1983; **18**: 82–92.
11. Yuan BY, Zhu RX and Tian WL *et al*. The age, subdivision and correlation of Nihewan Group. *Sci China Ser D* 1996; **26**: 67–76.
12. Pei SW, Li XL and Liu DC *et al*. Preliminary study on the living environment of hominids at the Donggutuo site, Nihewan Basin. *Chin Sci Bull* 2009; **54**: 3896–904.
13. Li XL, Pei SW and Jia ZX *et al*. Paleoenvironmental conditions at Madigou (MDG), a newly discovered early Paleolithic site in the Nihewan Basin, North China. *Quat Int* 2016; 400: 100–10.
14. Toth N. The Oldowan reassessed: a close look at early stone artefacts. *J Archaeol Sci* 1985; **12**: 101–20.
15. Clark JD. *Kalambo Falls Prehistoric Site, I: The Geology, Palaeoecology and Detailed Stratigraphy of the Excavations*. Cambridge: Cambridge University Press, 1969.
16. Leakey MD. *Olduvai Gorge, Vol. 3: Excavations in Beds I and II, 1960-63*. Cambridge: Cambridge University Press, 1971.
17. Isaac GL, Harris JWK and Kroll E. The stone artefact assemblages: a comparative study. In: Isaac G and Isaac B (eds.). *Koobi Fora Research Project, Volume 5: Plio-Pleistocene Archaeology.* Oxford: Clarendon Press, 1997, 262–99.
18. de la Torre I. The Early Stone Age lithic assemblages of Gadeb (Ethiopia) and the Developed Oldowan/early Acheulean in East Africa. *J Hum Evol* 2011; **60**: 768–812.
19. Xie F and Cheng SQ. Palaeoliths excavation in Cenjiawan Village, Yangyuan County, Hebei Province. *Acta Anthropol Sin* 1990; **9**: 265–72.
20. Xie F, Toth N and Schick K *et al*. The refitting study of Cenjiawan lithic artefacts unearthed in 1986. *Journal of Chinese Antiquity* 1994; **6**: 86–102.
21. Xie F, Li J and Liu LQ. *Palaeolithic Archeology in the Nihewan Basin*. Shijiazhuang: Huashan Literature & Arts Press, 2006.
22. Xie F and Li J. The application of lithic refitting method in the research of Cenjiawan site. *Journal of Chinese Antiquity* 1995; **7**: 25–38.
23. Cziesla E. On refitting of stone artefacts. In: Cziesla E, Eichoff S and Arts N *et al*. (eds.). *The Big Puzzle: International Symposium on Refitting Stone Artefacts, Studies in Modern Archaeology 1*. Bonn: Holos, 1990, 9–44.
24. Zhu RX, Potts R and Xie F *et al*. New evidence on the earliest human presence at high northern latitudes in northeast Asia. *Nature* 2004; **431**: 559–62.
25. Zhu RX, Hoffman KA and Potts R *et al*. Earliest presence of humans in northeast Asia. *Nature* 2001; **413**: 413–17.
26. Zuo TW, Cheng HJ and Liu P *et al*. Magnetostratigraphic dating of the Hougou Paleolithic site in the Nihewan Basin, North China. *Sci China Ser D* 2011; **54**: 1643–50.
27. Ao H, Deng CL and Dekkers MJ *et al*. Astronomical dating of the Xiantai, Donggutuo and Maliang Paleolithic sites in the Nihewan Basin (North China) and implications for early human evolution in East Asia. *Palaeogeogr Palaeoclimatol Palaeoecol* 2010; **297**: 129–37.
28. Li H, Yang X and Heller F *et al*. High resolution magnetostratigraphy and deposition cycles in the Nihewan Basin (North China) and their significance for stone artifact dating. *Quat Res* 2008; **69**: 250–62.
29. Tang YJ, Li Y and Chen WY. Mammalian fossils and the age of Xiaochangliang Paleolithic site of Yangyuan, Hebei. *Vertebrata Palasiatica* 1995; **33**: 74–83.
30. Li HM and Wang JD. Magnetostratigraphic study of several typical geologic sections in North China. In: Liu TS (ed.). *Quaternary Geology and Environment of China*. Beijing: Ocean Press, 1982, 33–8.
31. Schick KD and Zhuan D. Early Paleolithic of China and eastern Asia. *Evol Anthropol* 1993; **2**: 22–35.
32. Wang HQ, Deng CL and Zhu RX *et al*. Magnetostratigraphic dating of the Donggutuo and Maliang Paleolithic sites in the Nihewan Basin, North China. *Quat Res* 2005; **64**: 1–11.
33. Singer BS, Hoffman KA and Chauvin A *et al*. Dating transitionally magnetized lavas of the late Matuyama Chron: toward a new ^40^Ar/^39^Ar timescale of reversals and events. *J Geophys Res* 1999; **104**: 679–93.
34. Hilgen FJ, Lourens LJ and van Dam JA. The Neogene period. In: Gradstein FM, Ogg JG and Schmitz MD *et al*. (eds.). *The Geologic Time Scale 2012.* Amsterdam: Elsevier BV, 2012, 923–78.
35. Milankovitch M. *Canon of Insolation and the Ice Age Problem* (in Yugoslavian). Belgrade: Serb. Acad. Beorg. Special Publication, 1941. (translated by the Israel Program for Scientific Translations, Jerusalem, 1969).
36. Pisias NG and Moore TC. The evolution of Pleistocene climate: A time series approach. *Earth Planet Sci Lett* 1981; **52**: 450–8.
37. Maasch KA. Statistical detection of the mid-Pleistocene transition. *Clim Dynam* 1988; **2**: 133–43.
38. Short DA, Mengel JG and Crowley TJ *et al*. Filtering of Milankovitch cycles by Earth’s geography. *Quat Res* 1991; **35**: 157–73.
39. Clark PU, Archer D and Pollard D *et al*. The middle Pleistocene transition: Characteristics, mechanisms, and implications for long-term changes in atmospheric pCO_2_. *Quat Sci Rev* 2006; **25**: 3150–84.
40. Elderfield H, Ferretti P and Greaves M *et al*. Evolution of ocean temperature and ice volume through the mid-Pleistocene climate transition. *Science* 2012; **337**: 704–9.
41. Markova AK. Eastern European rodent (Rodentia, mammalia) faunas from the Early-Middle Pleistocene transition. *Quat Int* 2005; **131**: 71–7.
42. Head MJ and Gibbard PL. Early-Middle Pleistocene transitions: Linking terrestrial and marine realms. *Quat Int* 2015; **389**: 7–46.
43. Kitamura A. Constraints on eustatic sea-level changes during the Mid-Pleistocene Climate Transition: Evidence from the Japanese shallow-marine sediment record. *Quat Int* 2016; **397**: 417–21.
44. Ding ZL, Derbyshire E and Yang SL *et al*. Stacked 2.6-Ma grain size record from the Chinese loess based on five sections and correlation with the deep-sea δ^18^O record. *Palaeoceanography* 2002; **17**: 5-1–21.
45. Wang T, Sun YB and Liu XX. Mid-Pleistocene climate transition: Characteristic, mechanism and perspective. *Chin Sci Bull* 2017; **62**: 3861–72.
46. Mudelsee M and Schulz M. The Mid-Pleistocene climate transition: Onset of 100 ka cycle lags ice volume build-up by 280 ka. *Earth Planet Sci Lett* 1997; **151**: 117–23.
47. Medina-Elizalde M and Lea DW. The mid-Pleistocene transition in the tropical Pacific. *Science* 2005; **310**: 1009–12.
48. Zhu ZY, Dennell R and Huang WW *et al*. New dating of the *Homo erectus* cranium from Lantian (Gongwangling), China. *J Hum Evol* 2015; **78**: 111–57.
49. Shultz S, Nelson E and Dunbar RIM. Hominin cognitive evolution: Identifying patterns and processes in the fossil and archaeological record. *Philos T R Soc B* 2012; **367**: 2130–40.
50. Shen GJ, Gao X and Gao B *et al*. Age of Zhoukoudian *Homo erectus* determined with ^26^Al/^10^Be burial dating. *Nature* 2009; **458**: 198–200.
51. Grün R, Huang PH and Huang WP *et al*. ESR and U-series analyses of teeth from the palaeoanthropological site of Hexian, Anhui Province, China. *J Hum Evol* 1998; **34**: 555–64.
52. Wu XJ, Pei SW and Cai YJ *et al*. Archaic human remains from Hualongdong, China, and Middle Pleistocene human continuity and variation. *Proc Natl Acad Sci USA* 2019; **116**: 9820–4.
53. Yin GM, Bahain JJ and Shen GJ *et al*. ESR/U-series study of teeth recovered from the palaeoanthropological stratum of the Dali Man site (Shaanxi Province, China). *Quat Geochronol* 2011; **6**: 98–105.
54. Li ZY, Wu XJ and Zhou LP *et al*. Late Pleistocene archaic human crania from Xuchang, China. *Science* 2017; **355**: 969–72.
55. Li F, Bae CJ and Ramsey CB *et al*. Re-dating Zhoukoudian Upper Cave, northern China and its regional significance. *J Hum Evol* 2018; **121**: 170–7.
